# CAR-tropic extracellular vesicles carry tumor-associated antigens and modulate CAR T cell functionality

**DOI:** 10.1038/s41598-023-27604-5

**Published:** 2023-01-10

**Authors:** V. M. Ukrainskaya, O. E. Musatova, D. V. Volkov, D. S. Osipova, D. S. Pershin, A. M. Moysenovich, E. G. Evtushenko, E. A. Kulakovskaya, E. G. Maksimov, H. Zhang, Y. P. Rubtsov, M. A. Maschan, A. V. Stepanov, A. G. Gabibov

**Affiliations:** 1 M.M. Shemyakin and Yu.A. Ovchinnikov Institute of Bioorganic Chemistry of the Russian Academy of Sciences, Moscow, Russian Federation 117997; 2 Dmitry Rogachev National Medical Research Center of Pediatric Hematology, Oncology and Immunology, Moscow, Russian Federation 117997; 3 Faculty of Biology, Lomonosov Moscow State University, Moscow, Russian Federation 119991; 4 Faculty of Chemistry, Lomonosov Moscow State University, Moscow, Russian Federation 119991; 5 State Key Laboratory of Medicinal Chemical Biology and College of Life Sciences, Nankai University, 94 Weijin Road, Tianjin, 300071 China; 6 Blokhin National Medical Research Center of Oncology, Ministry of Health, Moscow, Russian Federation; 7 Department of Chemistry, The Scripps Research Institute, 10550 North Torrey Pines Road MB-10, La Jolla, CA 92037 USA; 8 Higher School of Economics, Moscow, Russian Federation 101000

**Keywords:** Cancer microenvironment, Targeted therapies

## Abstract

Tumor-derived extracellular vesicles (EVs) are active contributors in metastasis and immunosuppression in tumor microenvironment. At least some of the EVs carry tumor surface molecules such as tumor-associated antigens (TAAs) and/or checkpoint inhibitors, and potentially could interact with T cells or CAR T cells. Upon contact with T cells, EVs could alter their phenotype and functions by triggering signaling through TCR or CAR reprogramming them to escape immune response. We hypothesize that EVs that possess TAA on the surface will probably interact with CAR T cells which can recognize and bind corresponding TAA. This interaction between EVs and CAR T cells may change the outcome of CAR T-based cancer immunotherapy since it should affect CAR T cells. Also, EVs could serve as adjuvants and antigenic components of antitumor vaccines. Herein, we isolated EVs from B cell precursor leukemia cell line (pre-B ALL) Nalm-6 and demonstrated that recognition and binding of CD19^+^EVs with CD19-CAR T cells strongly depends on the presence of CD19 antigen. CD19^+^EVs induce secretion of pro-inflammatory cytokines (IL-2 and IFN-y) and upregulated transcription of activation-related genes (IFNG, IFNGR1, FASLG, IL2) in CD19-CAR T cells. Tumor necrosis factor receptor superfamily (TNFRSF4 and TNFRSF9) and T-cell exhaustion markers (CTLA4, LAG3, TIM3 and PDCD1LG2) were also upregulated in CD19-CAR T cells after incubation with CD19^+^EVs. Long-term cultivation of CD19^+^ or PD-L1^+^EVs with CD19-CAR T cells led to increased terminal differentiation and functional exhaustion according to elevated expression of PD-1, TIGIT, CD57. In summary, our results suggest that chronic exposure of CD19-CAR T cells to CD19^+^EVs mediates activation and systemic exhaustion in antigen-specific manner, and this negative effect is accompanied by the impaired cytotoxic activity in vitro.

## Introduction

Adoptive cell therapy with chimeric antigen receptor (CAR) modified T cells is one of the actively developing strategies in cancer therapy^1^. Striking efficacy of treatment of adult relapsed or refractory lymphomas and pediatric relapsed acute lymphoblastic leukemia by CAR T cells specific to the abundant B-cell marker CD19 was confirmed by numerous clinical trials^2,3^. CARs are recombinant receptors containing ligand-binding domain (typically a scFv), extracellular spacer, a transmembrane domain and intracellular signaling domains consisting of one or two co-stimulatory domains (typically CD28 or 4-1BB), and an activation domain (often CD3ζ). In contrast to T cell receptor (TCR) which engages HLA-peptide complexes, the binding domain of CAR interacts with a target molecule that does not require peptide processing or HLA expression to be recognized. After infusion, in response to tumor-associated antigen (TAA) CAR T cells become activated and rapidly expand to large numbers.

The patient’s tissues significantly differ from supportive environment during CAR T cell manufacturing or conditions used in in vitro and in vivo testing. One of the important factors that has to be considered is circulating tumor-derived extracellular vesicles (EVs) originating from tumor cells that could negatively affect functions of immune cells, and thus inhibit antitumor immunity^4–6^. Recent studies highlight that EVs are an important component of the immunosuppressive tumor microenvironment and have a significant impact on tumor progression^7–9^. It should be emphasized that tumor cells secrete more vesicles in comparison to cells of the surrounding tissue, which can be attributed to the fact that they proliferate rapidly under constant stress conditions^7,10,11^. EVs are small (< 1 μm in size) vesicles that likely transfer DNA, RNA, lipids, proteins, and metabolites from cell to cell^12–15^. Also EVs can carry membrane proteins from a parent cell. As a result, EVs may expose TAAs and/or checkpoint inhibitors on their surface. Presence of markers targeted by the CAR on the EVs theoretically will result in the preferable binding of EVs to CAR expressing cells over others. We questioned whether TAAs presented on the surface of EVs could affect the outcome of CAR T cell immunotherapy.

Previously, we and others demonstrated that CD19-positive vesicles can stimulate and ameliorate CAR T cells^16^ or have a negative effect on functionality of T or CAR T cells^17–19^. Here, we showed that recognition and binding of EVs with CD19-CAR T cells relies on EVs surface antigens. The binding of CD19^+^EVs to CD19-CAR T cells has high affinity and specificity due to antibody-antigen interactions and therefore is strikingly different from previously reported mechanisms of sticking and fusion of EVs to T cells^19,20^. After investigating phenotypic and functional consequences of CAR T cells exposed to EVs, we found that EVs may impact CAR T cells in a different way depending on vesicle concentration or set of surface target proteins.

## Results

### Isolation and characterization of tumor-derived extracellular vesicles

**Figure 1:**
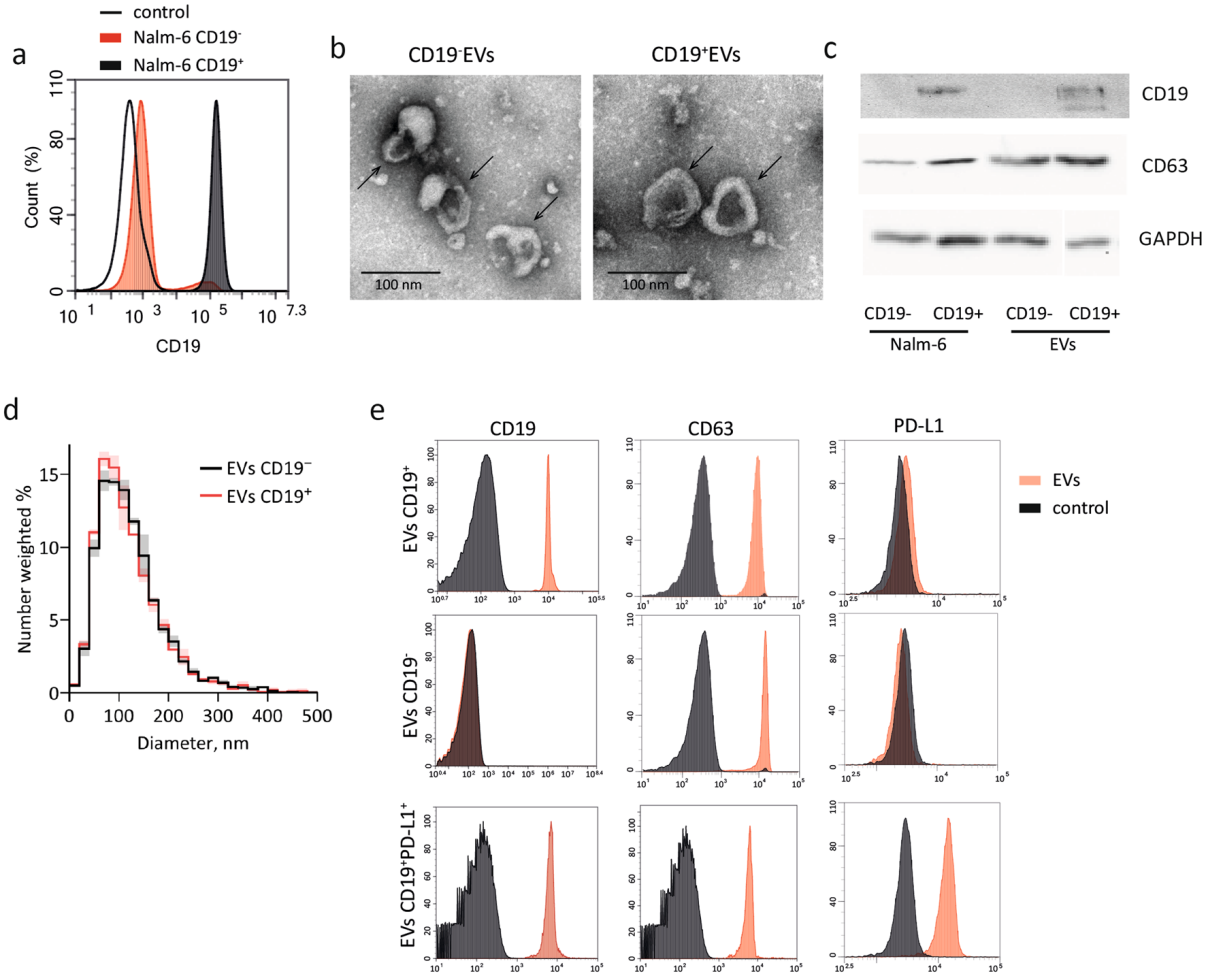
Characterization of EVs isolated from tumor cells. (**A**) CD19^+^ and CD19^−^ Nalm-6 cells were stained with anti-CD19 antibody conjugated with PE-Cy7 and analyzed by flow cytometry. Results are plotted as histograms. Background fluorescence of unstained control cells is shown as black line. (**B**) Cropped TEM images of purified CD19^+^ and CD19^−^ EVs. Original images are presented in Supplementary Figure 6B. (**C**) Western blot analysis of CD63 and CD19 expression in Nalm-6 total protein extract and extracts prepared from samples of purified EVs. Cropped images are presented. Aliquots of protein extracts were separated by SDS-PAGE, GAPDH was used for loading control and normalization. Original blots/gels are presented in Supplementary Figure 6A. (**D**) Histograms of size distribution of isolated EVs determined by NTA. Solid lines show mean values and shading represent SD for two independent samples. (**E**) Detection of EVs surface protein markers (CD19, CD63, PD-L1) on EVs captured on the surface of magnetic beads conjugated with anti-CD81 IgGs. Grey histograms show fluorescence of control empty beads stained with corresponding antibodies.

To evaluate the pattern of cellular markers exposed by tumor-derived extracellular vesicles (EVs), we collected supernatant from the pre-B cell lymphoma cell line Nalm-6 and isolated EVs. Purified EVs were analyzed for expression of 37 exosomal proteins. As expected, pre-B ALL-derived EVs carry tetraspanins (CD9, CD81, and CD63), several forms of integrins and MHC molecules (Supplementary Figure 1). Additionally, we discovered that Nalm-6 EVs are exposing CD19 and CD20—antigens used as targets of CAR molecule in CAR T therapy of B-cell malignancies (Supplementary Figure 1). Then, to study the contribution of antigen-specific interaction, we knocked-out CD19 gene in Nalm-6 to get Nalm-6 CD19^−^ cell line for CD19^−^ EVs production of EVs lacking CD19**—**CD19^−^ EVs (Fig. 1A; Supplementary Figure 2A). The juxtaposition of CD19^+^ and CD19^−^ EVs produced by Nalm-6 should allow excluding the influence of other exosomal components on the CD19-CAR T cells. This stringent control contrasts with common practice of using exosomes isolated from different cell line lacking CD19 expression as negative control (Fig. 1A).

EVs from both CD19^+^ and CD19^−^ Nalm-6 cells were isolated from culture medium conditioned by tumor cells by precipitation with ultracentrifugation. Transmission electron microscopy (TEM) was used to confirm the shape, quality and purity of EVs. TEM images demonstrate the presence of cup-shaped EVs in samples following ultracentrifugation that matches previously reported data^21,22^ (Fig. 1B).

Nanoparticle tracking analysis of isolated EVs revealed that their hydrodynamic diameter ranged between 60 and 200 nm with a mean around 120 nm which is typical for extracellular vesicles (Fig. 1D). Total particle concentration ranged from 2.5 × 10^11^ to 1.4 × 10^12^ particles/mL. Next, we analyzed the expression of tetraspanins CD81, CD63 and pan B-cell marker CD19 using immunostaining followed by flow cytometry or Western Blot. As expected, CD81 and CD63 were presented in all samples of EVs irrespectively of CD19 expression (Fig. 1C, E). Meanwhile, CD19 protein was detected on EVs isolated from CD19^+^ cells, but not on the EVs from CD19^-^ Nalm-6 (Fig. 1C, E).

Recent studies highlight the importance of immune checkpoint inhibitors presented on some EVs on dysfunction of CAR T cells^23,24^. We hypothesized that CAR-tropic vesicles with PD-L1 should be even more immunosuppressive for CAR T cells. Therefore Nalm-6 were stably transduced with lentiviral particles coding for PD-L1 (Supplementary Figure 2B) and used for isolation of CD19^+^PD-L1^+^EVs to study the effect of immune checkpoint inhibitors on activity of CD19-CAR T cells (Fig. 1E).

### CD19-CAR T cells specifically bind and uptake CD19^+^EVs

To track extracellular vesicles Nalm-6 CD19^−^ and glioblastoma U87 cell lines were transduced with lentiviruses coding for two exosome-specific reporter proteins—tetraspanin CD63 fused with nanoluciferase (CD63-nluc) or eGFP (CD63-eGFP) (Supplementary Figure 2C; Supplementary Figure 3A)^25^. These reporter vesicles are convenient tool for exploring the specificity and dynamics of interactions between EVs and acceptor cells. The amounts of CD63-nluc EVs are proportional to the luminescence signal, and their binding or uptake by target cells can be measured quantitatively. To determine whether the luminescent signal of the conditioned media originates from EVs, we compared the luciferase activity prior to and following concentration by ultracentrifugation. Samples of concentrated EVs had much stronger luminescence than initial culture media (Supplementary Figure 2D).

**Figure 2:**
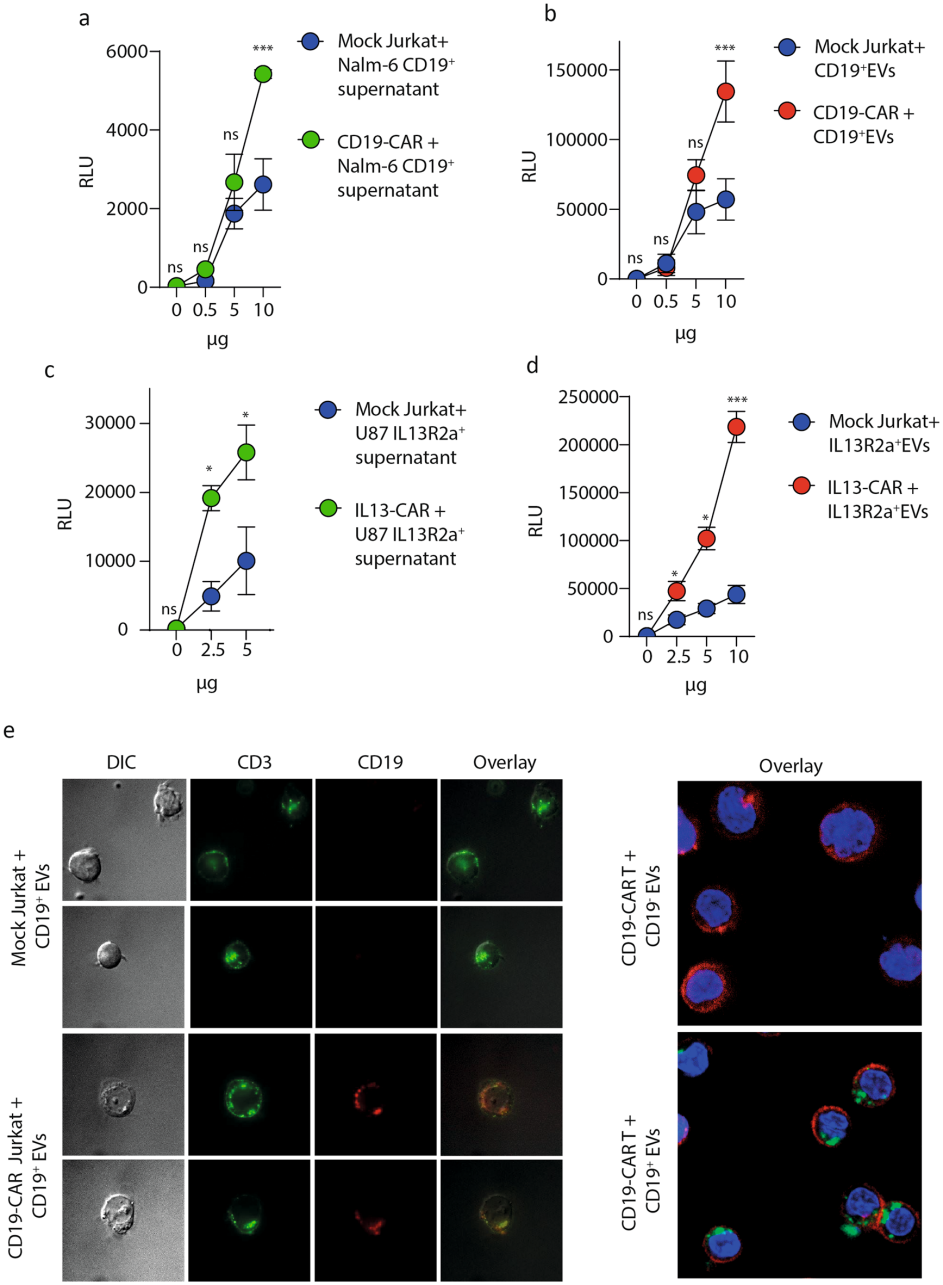
Specific uptake of CD19^+^ EVs by CD19-CAR T cells. The measurement of luciferase signal from CAR Jurkat cells treated with different concentration of supernatant from cultures of CD63-nluc Nalm-6 cells (**A**), or different doses of CD19^+^EVs (**B**). Level of nluc activity was assessed 10 min post-addition of supernatants or EVs. Data are presented as the mean ± SD of three experimental replicates obtained in two independent experiments. Statistical analysis was performed using two-way ANOVA with Tukey’s multiple comparison test. **C, D** The measurement of luciferase signal from CAR Jurkat cells treated with different concentration of supernatant from cultures of CD63-nluc Nalm-6 cells (**C**), or different doses of CD19^+^EVs (**D**). Level of nluc activity was assessed 10 min post-addition of supernatants or EVs. Data are presented as the mean ± SD of three experimental replicates obtained in two independent experiments. Statistical analysis was performed using two-way ANOVA with Tukey’s multiple comparison test. **E (left)** Confocal imaging of CD19-CAR Jurkat cells incubated with CD19^+^EVs isolated from Nalm-6 (**A**). Control Jurkat cells or CD19 CAR Jurkat cells were incubated with a suspension of CD19^+^EVs prior to microscopy. Jurkat cells were stained with APC anti-human CD3 mAb (green), EVs—with PE anti-human CD19 mAb (red). Representative images are shown. **E (right)** Confocal images of CD19-CAR T cells incubated with EVs, isolated from CD19^+^Nalm-6 and CD19^−^Nalm-6 (CD19^−^) with CD63-GFP. CD19-CAR T cells were incubated with a suspension of CD19^+^EVs or CD19^−^EVs prior to microscopy. Nuclei were stained with Hoechst 33342 (blue), T cells—with APC anti-human CD3 mAb (red), EVs labeled with CD63-GFP are green. Representative images are shown.

The uptake of purified CD19^+^EVs by CD19-CAR Jurkat cells was studied (Supplementary Figure 2E). CD19-CAR Jurkat cells were incubated with different amounts of EVs or culture media conditioned by Nalm-6 cells. The antigen-specific uptake was analyzed by assessment of the luciferase activity associated with CD19-CAR Jurkat. Mock Jurkat cells were used as a negative control. This assay revealed that the luciferase activity was significantly higher in samples of CD19-CAR Jurkat cells (Fig. 2A, B).

Next, we measured the vesicle uptake by IL13-CAR and Mock Jurkat cells incubated with EVs isolated from U87/CD63-nluc cells line expressing IL13R2a (Supplementary Figure 3B). Data presented in Fig. 2C, D demonstrate that the IL13R2a^+^EVs preferentially bind to the CAR-positive cells, indicating that interactions between CAR Jurkat cells and antigenic EVs rely on antigen and CAR specificity.

Confocal images of CAR Jurkat cells following incubation with EVs were obtained to determine whether EVs could be internalized by CAR Jurkat cells and explore the specificity of internalization. Microscopic images of CD19-CAR Jurkat cells are presented in Fig. 2E, left. The selective binding of CD19^+^EVs can be clearly seen only in the case of CAR-positive cells. The images of EVs-treated CD19-CAR T cells derived from T cells of healthy donor are shown in Fig. 2E, right. CD19-CAR T cells were incubated with EVs isolated from unmodified Nalm-6 CD63-eGFP (CD19^+^ EVs) or control Nalm-6 CD63-eGFP (CD19^−^ EVs) cells. eGFP-labeled EVs were detected only when CD19-CAR T cells were combined with CD19^+^ EVs (Fig. 2E, right), but not with control CD19^−^ EVs. These data provide additional evidence that vesicle uptake by CAR T cells is dependent on CAR specificity and target antigen on EVs.

### CD19^+^EVs activate CD19-CAR T cells

**Figure 3:**
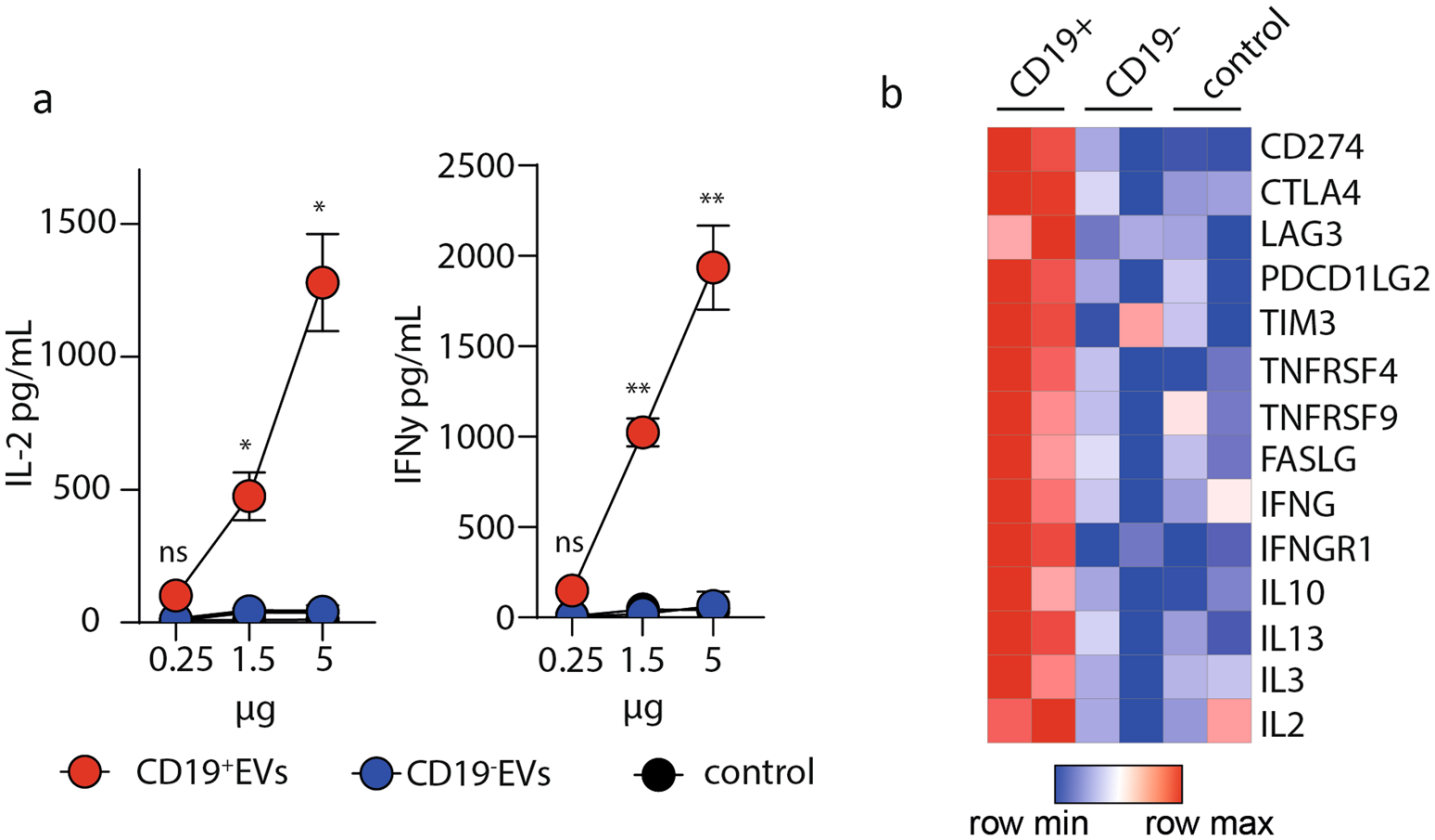
CD19-positive exosomes activate CAR T cells. **A** IFN-γ and IL-2 secretion by CD19-CAR T cells cultured in the presence of increasing concentrations of CD19^+^ or CD19^−^EVs. Analysis of IFN-γ and IL-2 secretion by CD19-CAR T cells in the absence of stimulation is shown as control. Data are presented as the mean ± SD of three experimental replicates in at least two independent experiments. Statistical analysis was performed using two-way ANOVA with Tukey’s multiple comparison test. **B** Targeted multiplex analysis of CD19-CAR T cells (n = 2) treated with CD19^+^ or CD19^−^ EVs. Untreated CD19-CAR T cells were used as a control. Heatmap represents genes with highest (red) and lowest (blue) expression (*p* value < 0.01, fold change > 1.5). Color key indicates the changes of normalized expression values.

Antigen recognition leads to the activation of CAR T cells and the release of pro-inflammatory cytokines. CD19-CAR T cells (32% CAR-positive cells, Supplementary Figure 2F) were co-cultured with various concentrations (0.25, 1 and 5 μg) of CD19^+^ and CD19^−^EVs for 24 h to evaluate the antigen-specific activation of CD19-CAR T cells in the presence of EVs. CD19^+^EVs induce activation of CD19-CAR T cells, which leads to the IFN-γ and IL-2 production. The level of pro-inflammatory cytokines directly correlated with amount of the CD19^+^EVs added to the CAR T cells. CD19^-^ EVs didn’t induce the activation of CD19-CAR T cells and cytokine release (Fig. 3A).

Further, we performed a transcriptomic analysis of CD19-CAR T cells co-cultured with CD19^+^ and CD19^−^ EVs for 24 h using NanoString RNA protocol (Fig. 3B). Untreated CD19-CAR T cells were used as negative control. We found the increased upregulation of activation-related genes (IFNG, IFNGR1, FASLG, IL2) only after treatment with CD19^+^EVs. RNA-seq data also demonstrated that expression levels of several members of tumor necrosis factor receptor superfamily (TNFRSF4 and TNFRSF9) and T-cell exhaustion markers (CTLA4, LAG3, TIM3 and PDCD1LG2) were upregulated dramatically after incubation with CD19^+^EVs. Together, these results demonstrate that cytokine release was strongly induced by CD19^+^EVs stimulation.

### CD19^+^ and PD-L1^+^EVs accelerate CD19-CAR T cells exhaustion

**Figure 4:**
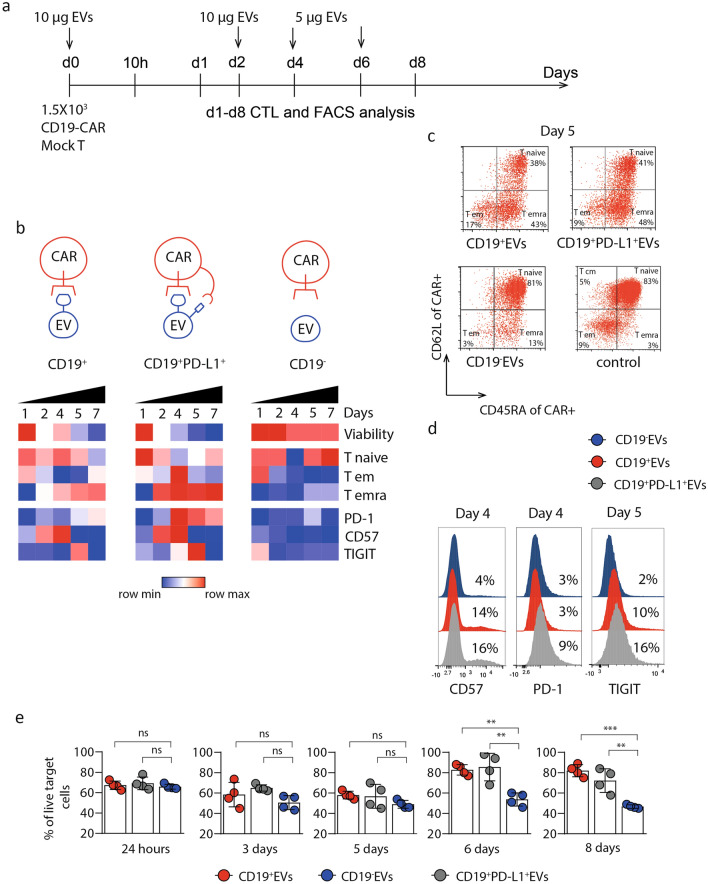
CD19^+^EVs accelerate exhaustion and impair CD19-CAR T cells’ killing activity. **A** Experimental timeline. **B** Changes in viability and surface phenotype of CAR T cells cultured in presence of CD19^+^, CD19^−^ or CD19^+^PD-L1^+^ EVs and analyzed daily for 7 days. Data were normalized against T cells treated with corresponding EVs and presented as heat map of the mean of two experimental replicates from two independent experiments. **C** Representative histograms show the difference in proportion of CAR T cells expressing markers of functional exhaustion: CD57 (left), PD-1 (center), TIGIT (right). CAR T cells stained for corresponding markers were analyzed by flow cytometry on days 4–5. **D** Analysis of differentiation state of CAR-positive cells cultivated for 5 days in the presence of EVs. CD19-CAR T cells were stained with anti-CD62L and anti-CD45RA mAbs and CD19-FITC conjugate, analyzed by flow cytometry, and plotted as two-dimensional dot plots. CAR T cells incubated for 5 days in the absence of EVs served as negative control. **E** In vitro killing activity at different time points indicated in Fig. 3A of CD19-CAR T-cells cultured for 20 h with Nalm-6 cells at 1:16 E:T ratio in the presence of CD19^+^, CD19^−^, CD19^+^PDL-1^+^EVs or without EVs. Data are presented as the mean ± SD of four experimental replicates from at least two independent experiments. Statistical analysis was performed using one-way ANOVA with Tukey’s multiple comparison test.

The therapeutic efficacy of CAR T cells directly correlates with the differentiation status of the modified T cells. Accumulation of terminally differentiated «exhausted» T cells associates with the poor prognosis and higher risk of tumor progression. CD19^+^EVs may chronically stimulate CAR and activate CAR T cells in the absence of target tumor cells that could potentially result in CAR T functional exhaustion and apoptosis. We analyzed the frequency of CAR T cells expressing exhaustion markers PD-1, TIGIT, CD57 stimulated with CD19^+^ or CD19^+^PD-L1^+^EVs. CD19-CAR T cells were cultivated in the presence of CD19^−^, CD19^+^ or CD19^+^PD-L1^+^ EVs, respectively, in the long-term experiment (Fig. 4A). Phenotypic analysis of CAR T cells (proportions of cells with different differentiation status, exhausted and viable cells) and assessment of cytotoxic function were performed from Day 1 to Day 8 according to the chart shown in Fig. 4A. Data were normalized using Mock T cells incubated with corresponding EVs as negative control. We observed substantial decrease in viability over time for CAR T cells cultured in the presence of CD19^+^EVs and CD19^+^PD-L1^+^ EVs (Fig. 4B, Supplementary Figure 4A). Moreover, the addition of CD19^+^ and CD19^+^PD-L1^+^ EVs resulted in increase in terminal differentiation of CAR-positive cells at Day 5, up to 43% (*p* = 0.007) and 48% (*p* = 0.004) of CAR + T_emra_ cells respectively, in comparison to 13% (*p* = 0.587) of CAR + T_emra_ cells following incubation with CD19^-^EVs (Fig. 4C). Frequencies of exhausted CAR T cells (according to surface markers) were also elevated in the samples treated with CD19^+^ and CD19^+^PD-L1^+^EVs. We observed at least several fold increase of CD57^+^ or TIGIT^+^ subpopulations on day 4 and day 5 after addition of EVs (Fig. 4D). Elevated proportion of PD-1^+^ CAR T cells was detected exclusively in the samples treated with CD19^+^PD-L1^+^ EVs (*p* = 0.019). Treatment of CAR T cells with EVs did not affect CD4/CD8 ratio according to experimental data (Supplementary Figure 4B).

### CD19^+^PD-L1^+^ EVs impair CD19 CAR T cells killing activity

Along with the incubation of CAR T cells with CD19^−^, CD19^+^ and CD19^+^PD-L1^+^ EVs, their capacity to kill target Nalm-6 CD63-GFP cells was determined in cytotoxic assay. After 10 h of incubation the decrease of cytotoxic activity of CD19^+^EVs treated CAR T cells was observed. After 24 h of incubation with target cells, the cytotoxicity was restored to initial value (Fig. 4E). After 6 days of co-cultivation of CAR T cells with CD19^+^EVs, the cytotoxic activity of CD19-CAR T decreased more than twice (Fig. 4E, Supplementary Figure 5). Cells treated with control CD19^−^EVs maintained stable cytotoxic activity over time.

**Figure 5:**
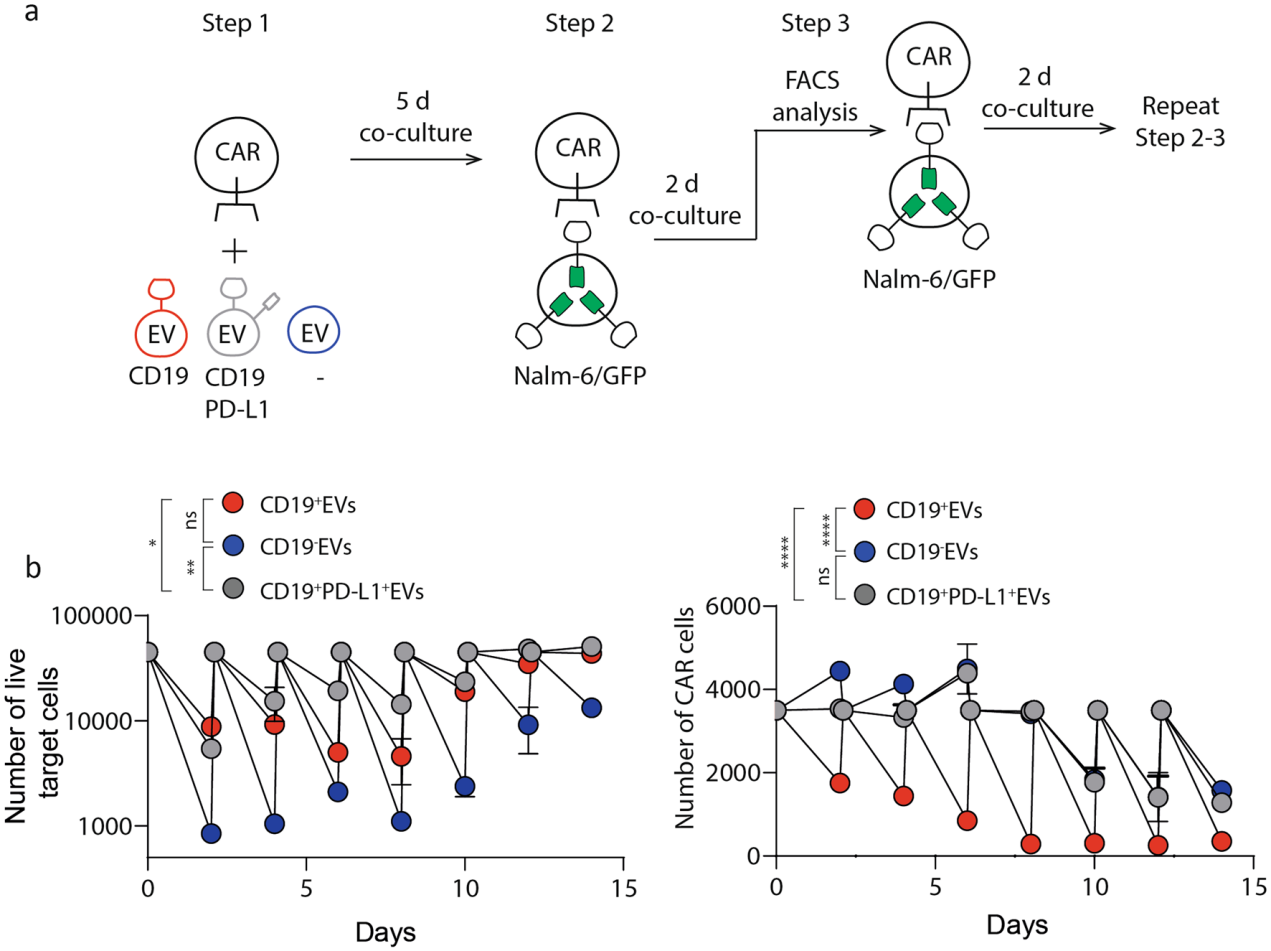
CD19-positive EVs impair killing of target cells by CD19-CAR T cells. **A** Design of sequential killing experiment. **B** Sequential killing assay of CD19^+^, CD19^+^PDL-1^+^, CD19^−^ EVs treated CD19-CAR T cells incubated with Nalm-6 target cells at 1:5 ratio. Target and CAR T cell numbers were analyzed every 2 days by flow cytometry. Remaining CAR T cells were mixed with fresh aliquot of target cells at the same ratio and incubated for another 2 days. The procedure was repeated several times until the day 14. Plots show number of survived target (left) and effector CAR T cells (right). Sequential killing assay started on the day 12 of co-cultivation of CARs with EVs. Data are presented as the mean ± s.d. of three experimental replicates and from at least two independent experiments. Statistical analysis was performed using two-way ANOVA with the Geisser-Greenhouse correction and Tukey’s multiple comparison test.

“Sequential killing” experiment was performed to assess proliferative potential of CAR T cells and the rate of exhaustion in the presence of EVs (Fig. 5A). CAR T cells pre-treated with EVs for 5 days were cultured with Nalm-6 CD63-GFP cells (E:T ratio 1:3) for several days. Several rounds of the cytotoxicity assay demonstrated that killing activity of CD19-CAR T cells was significantly reduced on day 6 and day 10 if they were co-cultured with CD19^+^PD-L1^+^ EVs and CD19^+^EVs respectively (Fig. 5B, left). The addition of CD19^−^ EVs causes the decrease of the CD19-CAR T cells killing activity only on day 15 of “Sequential killing” experiment (Fig. 5B, left). The proliferative activity of CD19^+^EVs treated CAR T cells was drastically reduced compared to the beginning of experiment, while CD19-CAR T cells cultured with CD19^+^PD-L1^+^EVs and CD19^-^EVs shows similar proliferation rate over time (Fig. 5B, right). Taken together, these data demonstrate the negative influence of CD19^+^EVs on proliferation, exhaustion and cytotoxic activity of CD19-CAR T cells in long-term co-cultivation experiments.

## Discussion

The hostile tumor microenvironment is considered to be the bottleneck of CAR T cell therapy in solid and diffuse tumors. Many factors of tumor microenvironment (TME) can lead to the CAR T cells dysfunction, but when it comes to diffuse cancers, circulating EVs play important role in suppression of the antitumor immune response. It is known that EVs can affect the functions and phenotype of recipient cells thereby regulating tumor progression and modulating the immune system^8^. Recent studies have shown that high level of circulating tumor-associated EVs may contribute to the lower effectiveness of therapy in CLL and AML patients^18,19^. EVs was proven to be an important factor of TME that inhibits T cell and CAR T anti-tumor activity both in vitro and in vivo^12^. The presence of PD-L1 on the surface of EVs has also been shown to cause suppression of both T cells and CAR T cells^19,23^.

We have investigated whether binding of EVs, bearing B-cell surface antigen CD19, to CAR could be one of the main reasons of CAR T cell dysfunction. The presence of CD19 on EVs is an important factor that can guide EVs to specific attachment to CD19-CAR T cells. The effect of CD19^+^ EVs on CAR T cells were compared with EVs isolated from Nalm-6 cells with ablated CD19 antigen to prove that specificity of CAR binding to its target and to minimize the potential impact of other EVs’ components on CAR T cells. We also generated Nalm-6 cells with PD-L1 overexpression to study the effect of PD-L1^+^ EVs on CAR-T cells. It is important to note that the PD-L1 expression rate is varying between the cancer types and individuals, thereby the application of artificially PD-1 overexpressing target cells could limit the interpretation of the results of this study. This antibody-antigen interaction leads to the specific attachment of CD19^+^EVs to the CAR-positive cells and as a result, the activation of CAR T cells in the absence of target cells. Moreover, such interactions are typical for different types of EVs carrying antigen, specific to CAR T cells. We demonstrate via RNA-seq profiling that this idle activation contributes to the chronic stimulation, upregulates a set of genes (CTLA-4, LAG3, TIM3 and PDCD1LG2), participating in functional maturation and subsequent exhaustion of CAR T cells. CD19-CAR T cell exhaustion was also characterized by terminal differentiation, upregulation of inhibitory receptors and senescence markers on the membrane of CD19-CAR T cells. Also, it was accompanied by markedly reduced CAR T viability. We observed gradual increase in proportion of CAR T cells positive for CD57 and TIGIT and, likewise, accumulation of T_em_ and T_emra_ CAR T cells during the first 5 days of experiment. However, at later time points, the difference in proportion of exhausted CD19-CAR T cells between samples treated with CD19^+^ and CD19^−^ EVs was not prominent. Possible explanation of this observation could be slow elimination of the exhausted and, therefore, hypoproliferative cells. We also discovered a moderate increase in PD-1 positive cells among CD19-CAR T cells treated with CD19^+^PD-L1^+^ EVs. The viability and exhaustion status of these cells were comparable with that of CAR T cells incubated with CD19^+^ EVs. The absence of strong effect could be explained by initially low frequency of PD-1 positive cells among CAR T cells prior to CD19^+^PD-L1^+^EVs addition.

Constant antigen stimulation results in phenotypically “exhausted”, yet still functional, CAR T cells^26,27^. To investigate the possible impact of CD19^+^EVs in CAR T cells’ killing activity we performed the long-term cultivation experiments. CD19-CAR T cells were assayed for the killing of CD19-positive Nalm-6 target pre-B ALL cells. During the first days of experiment there were no detectable changes in killing by CAR T, but at day 6 CD19-CAR T cells cultured with CD19^+^ and CD19^+^PDL-1^+^ EVs showed significant decrease of cytotoxic activity that correlates with gradual accumulation of CAR T cells with exhausted phenotype and reduction in the CAR T cells viability. Similar results were obtained in «sequential killing» assay, which revealed that CD19^+^EVs’- treated CD19-CAR T cells have the lowest killing capacity.

Finally, we did not evaluate the impact of EVs cargo on CAR T cells. It is suggested that EVs could deliver inhibitory signals to T cells by surface ligand-receptor interactions (Fas/FasL, PD-1/PD-L1, adenosine/ADORA2A) or transfer of nucleic acids (microRNA, mRNA)^5^. The preferable attachment of CD19^+^EVs to CAR T cells may facilitate the delivery of inhibitory signals from EVs to CAR T cells. We demonstrated that CD19^+^ tumor-derived EVs accelerate CAR T cell «exhaustion» and elimination accompanied by the loss of cytotoxic function. We suppose that these negative effects may be a likely reason for frequent relapses in diffuse cancers. In addition, the chemotherapeutic drugs may be added to EVs cargo and, as a result, may affect not only tumor cells, but also CAR T cells, in the course of combined CAR T plus chemotherapy treatment.

Taken together, the antigen-positive EVs induce in antigen-specific manner the dysfunctional CAR T cell phenotype and impair their killing activity. This functional inactivation of CAR T by EVs could be a reason for systemic exhaustion of CAR T cells in the immune suppressive tumor microenvironment.

## Materials and methods

### Cell culture

Nalm-6, U87 and Jurkat cell lines with confirmed free of mycoplasma contamination were provided by the Bioresource Collection—Collection of SPF-Laboratory Rodents for Fundamental, Biomedical and Pharmacological Studies supported by the Ministry of Science and Higher Education of the Russian Federation (Contract № 075-15-2021-1067). Nalm-6 were cultured in RPMI-1640 (HyClone, Logan, UT) containing 10% FBS (Sigma-Aldrich), 100 U/mL penicillin, and 100 mg/mL streptomycin, at 37 °C with 5% CO_2_. U87 were cultured in DMEM (HyClone, Logan, UT) containing 10% FBS (Sigma-Aldrich), 100 U/mL penicillin, and 100 mg/mL streptomycin, at 37 °C with 5% CO_2_. Two reporter lines were generated by transduction of Nalm-6 and Nalm-6 CD19^-^ cell lines with lentiviruses encoding CD63-GFP or CD63-nanoluc (Supplementary Figure 2G). The gene of CD63-GFP were cloned into the pLV2 lentiviral vector (Clontech) under control of EF1a promoter. The nucleotide sequence of CD63-nluc was cloned into the pCDH115 lentiviral vector under CMV promotor into dual-promoter lentiviral vector pCDH115 (with copGFP under control of the EF1a promoter).

The level of transduction was assessed by flow cytometry (ACEA Biosciences, USA). Enrichment of CD63-GFP and CD63-nanoluc positive cells was performed by sorting GFP-positive cells on the SH800 Cell Sorter (Sony, Japan).

### Nalm-6 CRISPR-Cas9 CD19 gene knockout

Two guide RNAs (gRNA) specific to the signal peptide of the human CD19 gene (GGCTCATGAGCTTCCCGGAA and GGGCGGGGACTCCCGAGACC) were utilized for Nalm-6 gene editing. Primers were synthesized according to the requirements of the sgRNA—HiScribe Quick T7 High Yield RNA Synthesis Kit (NEB, UK). Short DNA sequences were assembled by PCR and purified using the AMPure XP kit (Beckman, USA). PCR products were used for transcription using the HiScribe Quick T7 High Yield RNA Synthesis Kit (protocol for short transcripts). The resulting gRNA was purified using the AMPure XP kit, treated with DNase I, and the concentration was estimated on Qubit (Thermo Fisher, USA). For CD19 gene knockout 2 μg of gRNAs were mixed with 3.3 μL of Spy Cas9 NLS (BioLabs, England) and incubated for 30 min at room temperature. Then, 1.5 × 10^6^ Nalm-6 cells were washed twice with PBS, resuspended in 100 μL of electroporation buffer according to the protocol of P3 Primary Cell Solution Box (Lonza, Switzerland). Lonza 4D Nucleofector (Lonza, Switzerland) and EH100 program were used for electroporation. The effectiveness of the knockout was evaluated 5 days after electroporation. The CD19-negative cells were enriched by sorting on the SH800 Cell Sorter instrument (Sony, Japan).

### Extracellular vesicles isolation

Tumor cell lines were seeded at 2–2.5 × 10^6^/mL in RPMI/0.5% (vol/vol) BSA and cultured for 48 h at 37 °C, 5% CO_2,_ and EVs were harvested as described earlier with slight modifications^14,28^. Briefly, conditioned medium was aspirated and centrifuged at 300*g* for 10 min. Larger EVs were removed from the supernatant by two steps of centrifugation (2000*g* for 20 min and 12,000*g* for 30 min). All centrifugation steps were carried out at 4 °C. Cleared supernatants were filtered through 0.2-μm membrane filters (Thermo Fisher Scientific, USA). Smaller EVs were collected by ultracentrifugation at 100,000*g* for 90 min at 4 °C using Beckman L7-65 Ultracentrifuge equipped with SW 40 Ti rotor, USA. The resulting pellets, containing EVs, were resuspended in PBS and concentration of vesicles were determined using BCA assay kit (Thermo Fisher Scientific, USA).

### Western blot

Nalm-6 cell lysates or EVs from Nalm-6 cells were mixed with 2 × sample buffer (125 mM Tris·HCl pH 6.8, 4% (wt/vol) SDS, 20% (vol/vol) glycerol, 100 mM DTT, 0.01% bromophenol blue), and heated at 90 °C for 10 min. Proteins were separated on a 10% Mini-PROTEAN TGX gel (Bio-Rad, USA) and transferred to a nitrocellulose membrane (Bio-Rad, USA). CD63 was detected with rabbit anti-human CD63 HRP antibody (System Biosciences, USA). GAPDH was detected with anti-GAPDH antibody (Abcam, USA). CD19 was detected with mouse anti-human CD19-HRP antibody (Abcam, USA) (Supplementary Table 1, Supplementary Figure 6A).

### NTA analysis

The concentration of isolated EVs, as well as the particle size distribution, were determined by nanoparticle tracking analysis using a NanoSight LM10-HS instrument (Malvern Panalytical Ltd, UK) equipped with a 405 nm, 65 mW laser unit with passive temperature reading and a highly sensitive Andor Luca EMCCD camera. (Andor, Belfast, UK) (Supplementary Figure 6B). All measurements were performed in accordance with ASTM E2834-12 (2018) with camera and video processing settings optimized for EVs as previously described^29,30^. Each sample was diluted to a concentration of (1.3–2.0) × 10^8^ particles/ml, optimal for measurements. Twelve videos (60 s each) were recorded and processed using NTA 2.3 build 33 software (Malvern Panalytical Ltd, UK). All measurements (5200–6500 tracks total) were pooled to produce a histogram of particle size and total particle concentration corrected for dilution factor.

### Transmission electron microscopy

Transmission Electron Microscopy (TEM) was used to visualize the isolated EVs. The carbon-coated TEM grids (Ted Pella, USA) were pre-treated using an Emitech K100X device (Quorum Technologies, UK). Samples were applied onto the grids at a total protein concentration of 50–100 ng/μL and incubated for 3 min. Next, the grids were blotted, treated with 1% uranyl acetate solution, blotted again and dried. The images were obtained using a JEM-1400 electron microscope (Jeol, Japan) operating at 120 kV.

### Detection of chemiluminescence of CD63-nanoluc EVs

To carry out the reaction of oxidation of the substrate, a kit for the detection of chemiluminescence Nano-Glo Luciferase Assay (Promega, USA) kit was used to measure nanoluciferase activity. Suspension of EVs CD63-nanoluc or EVs treated CAR Jurkat cells were mixed in a 1:1 ratio with the Nano-Glo substrate and analyzed on a VarioScan instrument (Thermo Fisher, USA). All measurements were carried out in at least 5 repeats with corresponding negative controls to determine the background signal values.

### CAR T cell generation

The nucleotide sequence of the CD19-CAR^31^ consists of the following parts: anti-CD19 scFv (FMC63), CD8-α hinge region, 4-1BB co-stimulatory domains, and CD3ζ (signaling domain). The CAR19 gene was synthesized (GeneCust) and subcloned into the pLV2 lentiviral vector (Clontech) under control of the EF1a promoter.

The human IL13 mutein (E13Y) sequence was synthesized (GeneCust) and inserted into pLV2 lentiviral vector (Clontech) with third-generation CAR construct consisted of human IgG4-CH2CH3 domain, CD28 transmembrane domain, and costimulatory domains of CD28, OX-40, and the CD3ζ.

### Isolation and transduction of T cells

The lentiviral particles were prepared by PEI co-transfection of HEK293T cells with the corresponding lentiviral CAR plasmids and the packaging plasmids (p-VSV-G, pGAG and pREV). The lentiviral supernatants were collected 48 h after transfection. Peripheral blood of patients and healthy donors was obtained from Dmitry Rogachev National Research Center of Pediatric Hematology, Oncology and Immunology. Dynabeads Untouched Human T Cells Isolation Kit (Invitrogen, USA) was utilized for isolation of T cells from human PBMCs. Human T cells were activated with CD3/CD28 Dynabeads (Thermo Scientific, USA) at a 1:1 ratio (Life Technologies, USA) in a complete RPMI containing 40 IU/mL recombinant IL-2 (Pan Biotech, USA) for 24 h. Activated T cells were re-suspended at concentration of 0.5 × 10^6^/mL in lentiviral supernatant of CD19-CAR 1 mL of fresh RPMI media supplemented with 30 IU/ml IL-2 was added, and cells were plated to 6-well plates. Plates were centrifuged at 1200*g* for 90 min at 32 °C and incubated for 6 h at 37 °C. Then culture medium was changed every 2 days and cells were grown in flasks at a density of 2.0 × 10^6^/mL.

### Incubation of CAR Jurkat cells with EVs CD63-nanoluc

Supernatant of Nalm-6/CD63-nanoluc cells were concentrated on a 100-kDa Amicon membrane (Millipore, USA). Further, the supernatant was added to the 0.2 × 10^6^ CD19-CAR Jurkat and control Jurkat cells at various concentrations and incubated at 37 °C for 10 min. The cells were pelleted, washed before measurement of the nanoluciferase activity. A similar experiment was carried out with purified EVs.

### Confocal microscopy

0.5 × 10^6^ CAR T cells/Mock T or CAR Jurkat cells/Mock Jurkat were mixed with 10 μg CD19^+^EVs or CD19^-^EVs and incubated for 1 h at 4 °C. Then, cells were washed from unbound EVs (300*g*, 10 min 4 °C), plated on poly-L-lysine coated coverslips, and centrifuged (100*g*, 10 min, RT). Attached cells were fixed in 4% formalin for 1 h at room temperature and stained with anti-human CD3-APC (Biolegend, UK), Hoechst 33342 (Sigma, USA). The Nalm-6 EVs were detected with anti-human CD19-PE (Biolegend, UK) or by GFP fluorescence CD63-GFP EVs. Confocal imaging was performed at room temperature. Confocal images were acquired using an Axio Observer Z1 microscope (Carl Zeiss, Jena, Germany) with a Yokogawa rotating disc confocal device (CSU-X1, Yokogawa Corporation of America, Sugar Land, TX, USA).

### Analysis of cytokine release

For the analysis of cytokine release, 5 × 10^4^ CD19-CAR T cells mixed with freshly isolated Nalm-6 CD19^+^ or Nalm-6 CD19^-^ EVs in culture media without IL-2 and incubated for 24 h in a 96-well plate. As a negative control, CD19-CAR T cells were incubated without the addition of EVs. At the end of incubation, the supernatant was separated from the cells by centrifugation (4 °C, 300*g*, 5 min), transferred to a new plate. The concentration of IL-2 and IFNγ in the supernatants were analyzed using cytokine-specific ELISA kits (Vector-Best, Russia) in accordance with the manufacturer's instructions.

### EVs cytotoxicity assay for CAR T cells

The CD19-CAR T cells were mixed with 10 μg (10 μg of total protein) of CD19^+^ or CD19^−^ EVs and incubated at 37 °C and 5% CO_2_. At different time points (24 h, 3 days, 5 days, 6 days, and 8 days) the portion of CAR T cells were mixed with Nalm-6 GFP+ cells in various E:T ratios (1:4, 1:8, 1:16 and 1:32) and co-incubated for 20 h at 37 °C and 5% CO_2_ in RPMI 1640 complete medium supplemented with human IL-2 (30 IU/mL). The cytotoxicity of CAR T cells was analyzed by flow cytometry.

### Flow cytometry analysis

To characterize the pattern of surface antigens, EVs were immobilized on anti-CD81 beads according to the protocol of Exosome-Human CD81 Isolation kit (Invitrogen, USA). EVs attached to CD81+ beads were stained with anti-human CD19-PE (Biolegend, USA), anti-human CD63-APC (Sony, Japan), anti-human PDL-1-APC (Miltenyi, USA) anti-human CD81-FITC (Sony, Japan), anti-human IL13R2a-FITC (R&D systems, USA) and other antibodies described in Supplementary table 1 and analyzed by flow cytometry (ACEA Biosciences, USA). Flow cytometry was performed on NovoCyte 2060 (ACEA Biosciences, USA) and analyzed by FlowJo X10 (FlowJo) and NovoExpress Software (ACEA Biosciences, USA).

For multiplex analysis of Nalm-6-derivied EVs MACSPlex Exosome Kit (Miltenyi, USA) were used. Isolated EVs were incubated overnight with 39 differently labeled capture bead populations each coupled to a different antibody. After incubation with the APC-conjugated antibodies EVs were analyzed by flow cytometry (MACSQuant Analyzer 10, Miltenyi, USA).

For T cell exhaustion markers analysis the cells were stained with anti-human CD57-APC-Cy7 (Miltenyi, USA), anti-human PD-1-APC (Miltenyi, USA), anti-human TIGIT-PE (Miltenyi, USA), anti-human CD62L-APC (Sony, Japan), anti-human CD45RA-PE (Sony, Japan), FITC-Labeled Human CD19 (Acro Biosystems, USA) and other antibodies described in Supplementary table 1. The T and CAR T cells were incubated with monoclonal antibodies or isotype controls for 30 min at 4 °C, washed, and analyzed by flow cytometry.

### Gene expression analysis

The CAR positive population of transduced T cells were sorted out and 0.5 × 10^6^ of CAR T cells were mixed with 10 μg of CD19^+^ and CD19^−^EVs. After 24 h cells were washed from unbound EVs (300 g, 10 min 4 °C) and total RNA was extracted from CAR T cells using the RNeasy kit (QIAGEN GmbH, Hilden, Germany) following manufacturer's recommendations. RNA quality and concentration was determined using the NanoDrop spectrophotometer (NanoDrop Technologies, Oxfordshire, UK).

An aliquots of RNA samples (final concentration 100 ng/μL) were used for NanoString comparison analysis. Each gene of interest was detected using a pair of reporter and capture probes that together target a continuous 780 nucleotide sequence (CAR-T panel). Hybridization between target mRNA and reporter-capture probe pairs was performed at 65 °C for 16 h using CT1000 Touch Thermal Cycler (Bio-Rad, CA) according to the manufacturer's protocol. Post hybridization processing was carried out on a fully automated nCounter Prep station liquid-handling robot. Excess probes were removed, and the probe/target complexes were aligned and immobilized in the nCounter cartridge, which was then placed in a digital analyzer for image acquisition and data processing (nCounter Digital Analyzer, USA) as per the manufacturer’s protocol. The expression level of a gene was measured by counting the number of times the specific barcode for that gene was detected, and the barcode counts were then tabulated in a comma-separated value (CSV) format. The raw digital count of expression was exported from nSolver v3.0 software for downstream analysis (Supplementary Figure 7).

### Sequential killing assay

The CD19-CAR T cells pretreated with 10 μg CD19^+^, CD19^+^PD-L1^+^CD19^−^ EVs were mixed with 50 × 10^4^ Nalm-6/GFP target cells in 200 μL complete RPMI. To count the CAR T cells and target cells, we analyze samples by FC every 48 h. Nalm-6/GFP cells cultured without T cells were used as a reference. For the next cycle of sequential killing assay, the 10 × 10^4^ CAR T mixed with the fresh 50 × 10^4^ Nalm-6/GFP cells and incubated 2 more days. This procedure was repeated until there was complete disappearance of killing activity in one of the samples.

### Statistical analysis

All statistical analyses were performed using the Prism v8 (GraphPad Software). Each figure legend denotes the statistical test used. Mean values are plotted as bar graphs, error bars indicate SD unless otherwise stated. For all figures, * indicates *P* < 0.033, ** indicates *P* < 0.002, *** indicates *P* < 0.001, **** indicates *P* < 0.0005.

### Ethics statement

All experiments on human subjects were approved by the D. Rogachev center local ethics committee (decision on 18.01.2018) and were carried out in accordance with the approved protocols. The participants provided their written informed consent to participate in this study.

## Supplementary Information

Supplementary Information.

## Data Availability

The original contributions presented in the study are included in the article/Supplementary material. Further inquiries can be directed to the corresponding author.
