# Supplementary material for: CAR-tropic extracellular vesicles carry tumor-associated antigens and modulate CAR T cell functionality
Source: Sci Rep. 2023 Jan 10;13:463. doi: 10.1038/s41598-023-27604-5 (PMC9832131; doi:10.1038/s41598-023-27604-5)
Supplement: Supplementary file 1 — Supplementary Information. [file 41598_2023_27604_MOESM1_ESM.docx]

**Supplementary Material**

**CAR-tropic extracellular vesicles carry tumor-associated antigens and modulate CAR T cell functionality**

**Ukrainskaya V.M., Musatova O.E., Volkov D.V., Osipova D.S., Pershin D.S., Moysenovich A.M. , Evtushenko E.G., Kulakovskaya E.A., Maksimov E.G., Zhang H., Rubtsov Y.P., Maschan M.A., Stepanov A.V., Gabibov A.G..**


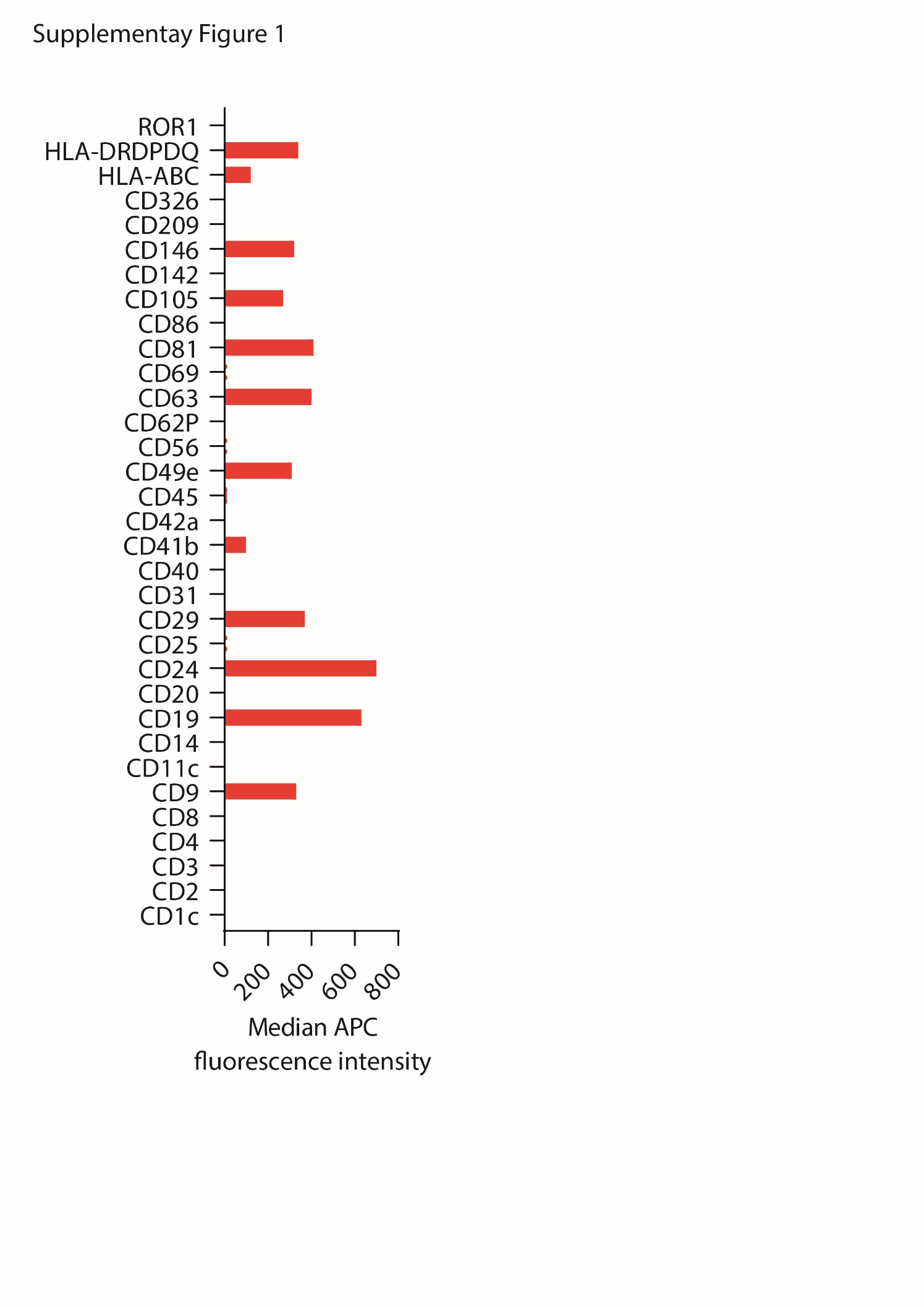


**Supplementary figure 1.** Characterization of the common lymphoma surface markers on Nalm-6-derived EVs.


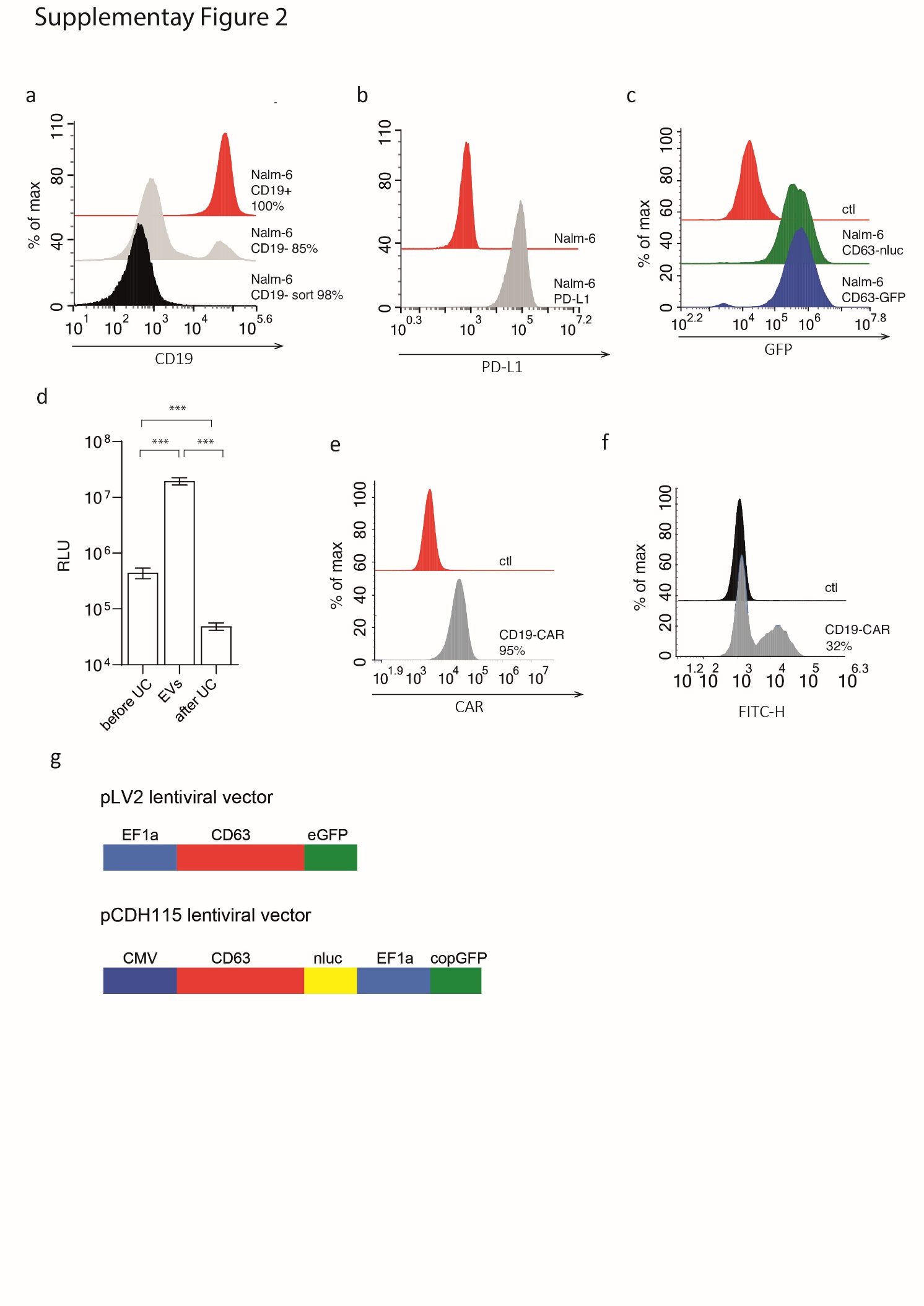


**Supplementary figure 2. Chemiluminescence assay of EVs samples and flow cytometry characterization of CAR T cells and tumor cell lines.** **A** - CD19^+^ and CD19^-^ Nalm-6 cells were stained with anti-CD19 antibody before and after FACS and analyzed by flow cytometry. Results are plotted as histograms. **B** - Nalm-6 cells positive for PD-L1 were stained with anti-PD-L1 antibody conjugated with APC and analyzed by flow cytometry. Results are plotted as histograms. Red histogram shows negative control. **C** - Nalm-6 reporter cells line Nalm-6 CD63-eGFP and Nalm-6 CD63-nluc were analyzed by flow cytometry. Results are plotted as histograms. Red histogram shows negative control. **D** – Analysis of nanoluc chemiluminescence in samples of EVs before and after UC, and in concentrated EVs sample. Data are presented as the mean ± SD. of six experimental replicates in at least two independent experiments. Statistical analysis was performed using t-test. **E,F** – Jurkat CD19-CAR and CD19-CAR T cells were stained with CD19 CAR detection reagent (Miltenyi, USA) followed by Streptavidin-FITC (Biolegend, USA) staining prior to flow cytometry. **G** – Lentiviral vectors encoded CD63-GFP and CD63-nluc proteins represented schematically.

**
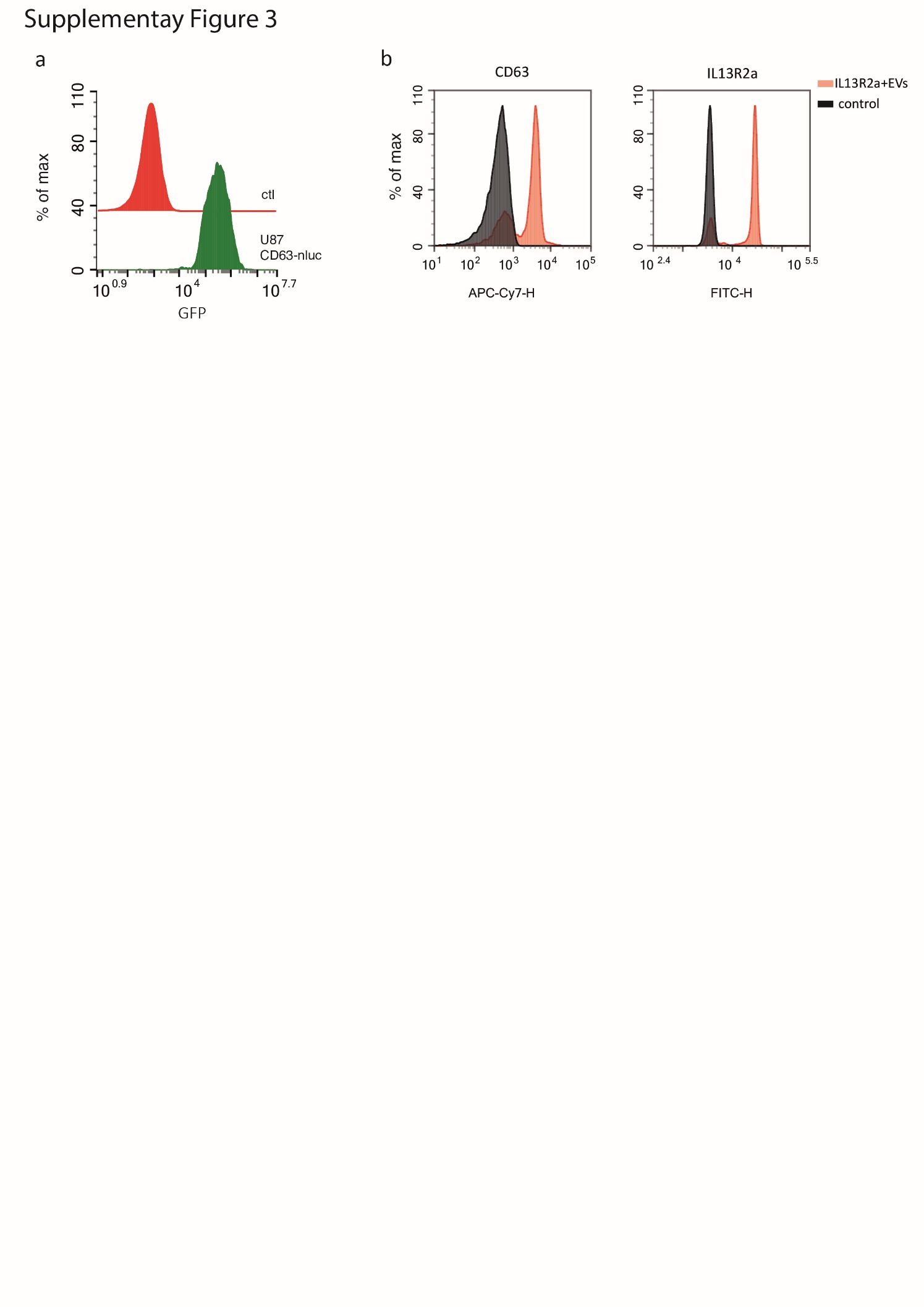
**

**Supplementary figure 3.** **Characterization of IL13R2a-positive EVs**. **A** – U87 CD63-nluc reporter cells line were analyzed by flow cytometry. Results are plotted as histograms. Red histogram shows negative control. **B** - Detection of IL13R2a^+^EVs surface protein markers (IL13R2a, CD63) on EVs captured on the surface of magnetic beads conjugated with anti-CD81 IgGs. Grey histograms show fluorescence of control empty beads stained with corresponding antibodies.


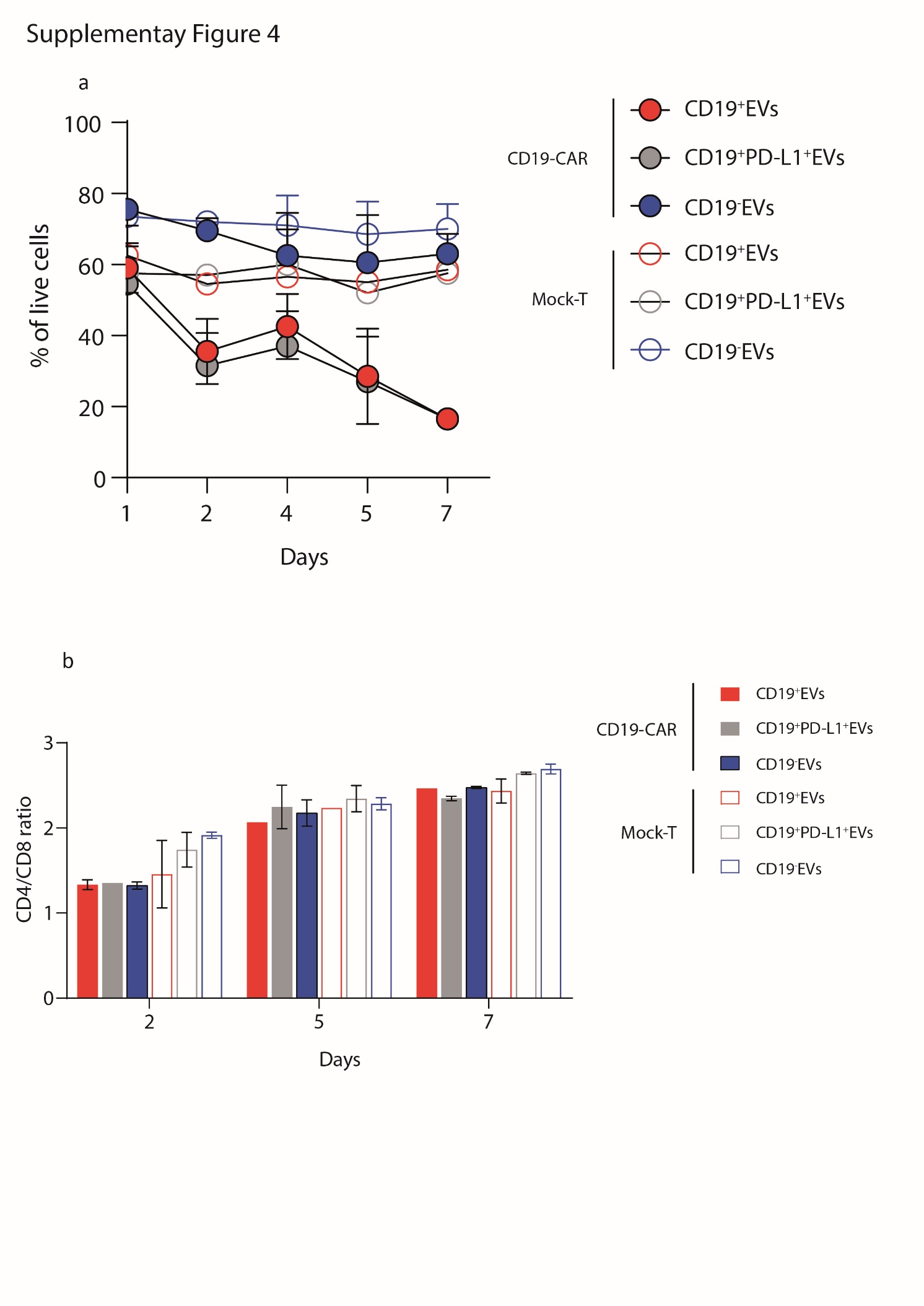

**Supplementary figure 4. Analysis of viability and CD4/CD8 ratio for tdEVs treated CD19-CAR T cells at different time points.** A - Changes in viability of CAR T cells and Mock T cells cultured in presence of CD19^+^, CD19^-^ or CD19^+^PD-L1^+^ EVs analyzed every day for 7 days. Data are presented as the mean ± SD of two experimental replicates in at least two independent experiments. B - Changes in CD4/CD8 ratio of CAR T cells and Mock T cells cultured in presence of CD19^+^, CD19^-^ or CD19^+^PD-L1^+^ EVs analyzed on day 2,5,7 of experiment. Data are presented as the mean ± SD of two experimental replicates in at least two independent experiments.


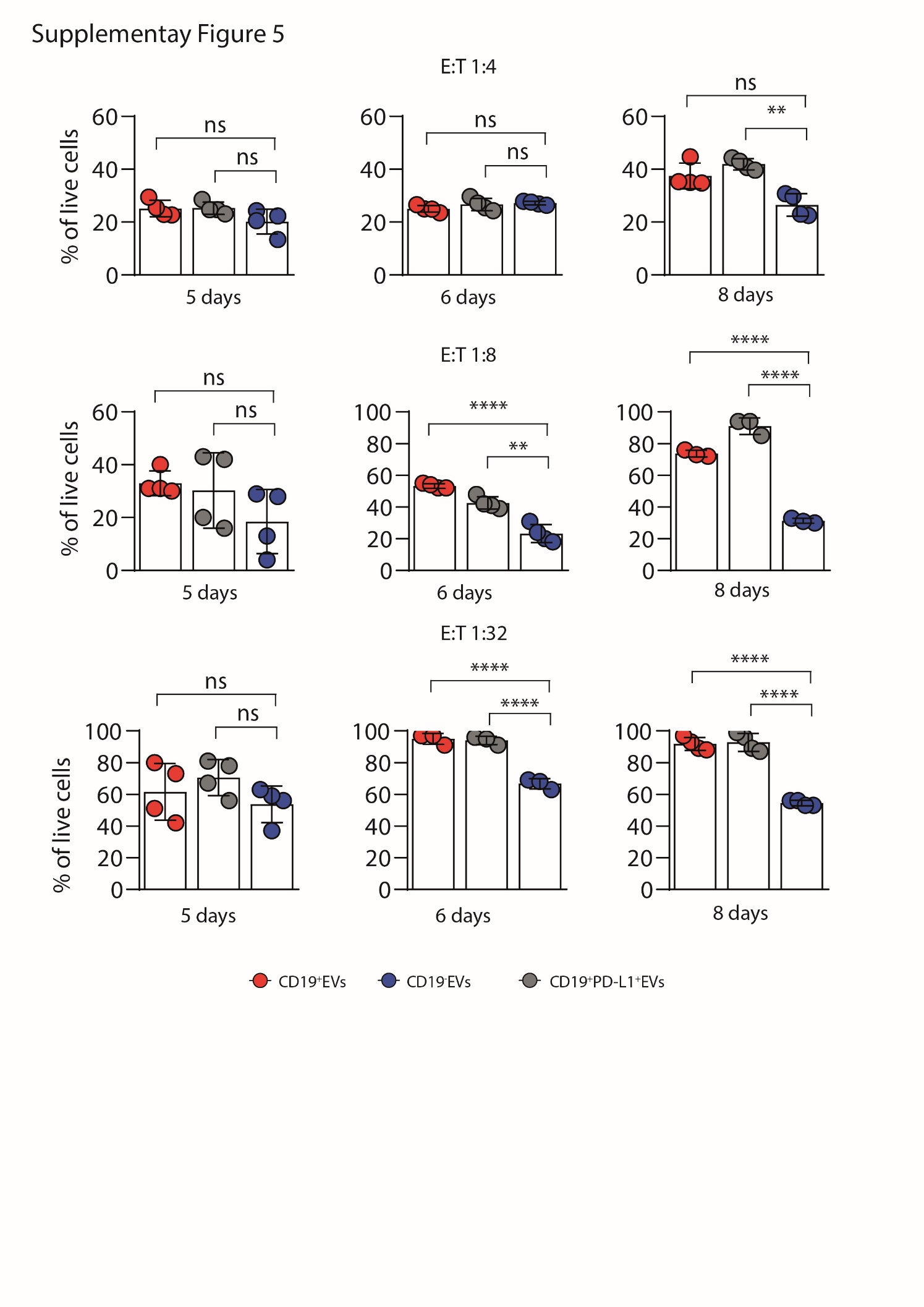


**Supplementary figure 5.** *In vitro* killing activity at different time points from fig 3A of CD19-CAR T-cells treated with CD19^+^, CD19^-^ or CD19^+^PD-L1^+^ EVs and cultured for 20 hours with Nalm-6 cells at 1:4, 1:8 and 1:32 E:T ratio. Data are represented as the mean ± SD of four experimental replicates from at least two independent experiments. Statistical analysis was performed using one-way ANOVA with Tukey’s multiple comparison test.


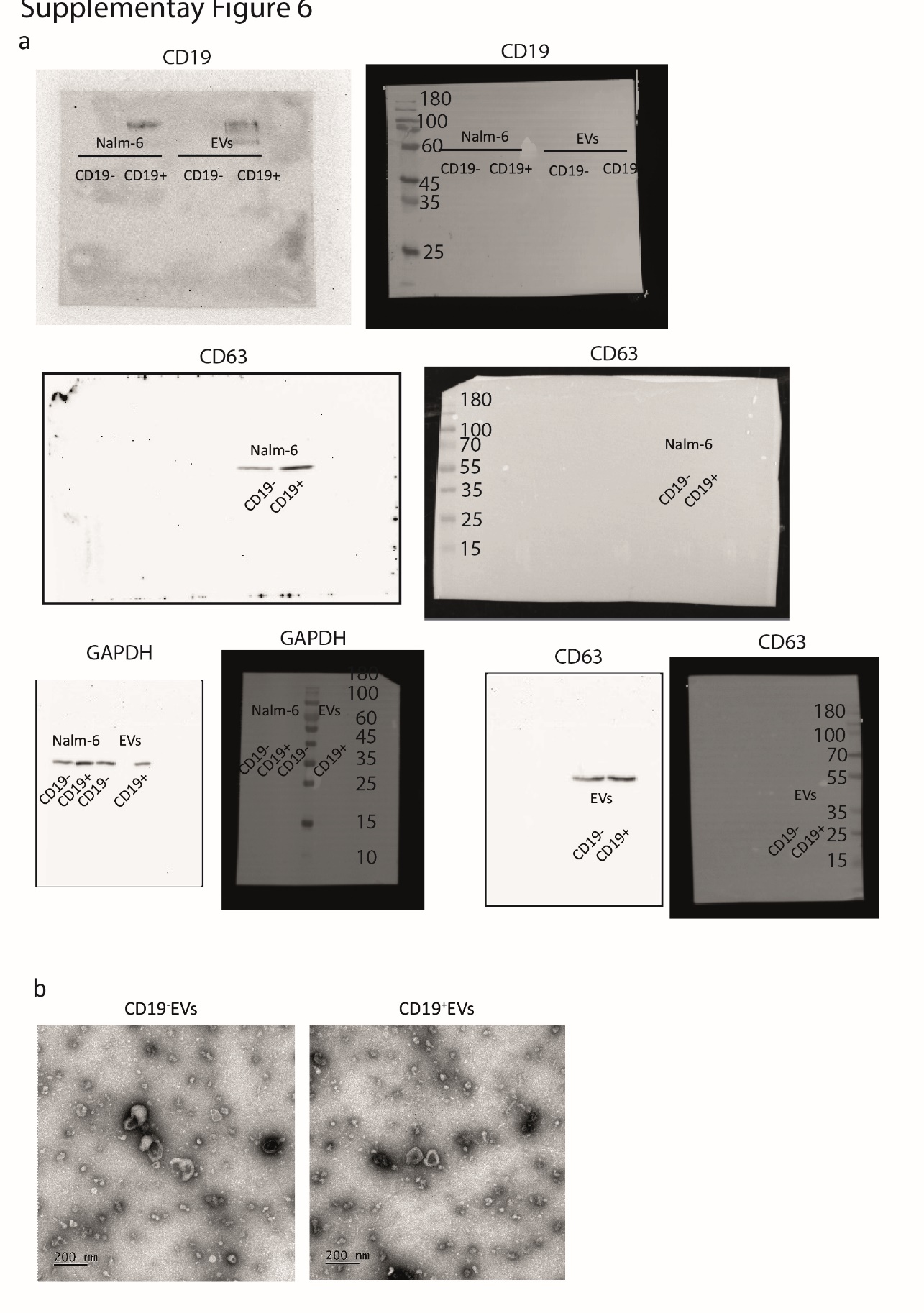


**Supplementary figure 6.** A – Western blot analysis of [CD63](https://www.sciencedirect.com/topics/biochemistry-genetics-and-molecular-biology/cd63) and [CD19](https://www.sciencedirect.com/topics/biochemistry-genetics-and-molecular-biology/cd9) expression in Nalm-6 total protein extract and extracts prepared from samples of purified EVs. Aliquots of protein extracts were separated by SDS-PAGE, GAPDH was used for loading control and normalization. Uncropped images are presented. B – Uncropped TEM images of purified CD19^+^ and CD19^-^ EVs.


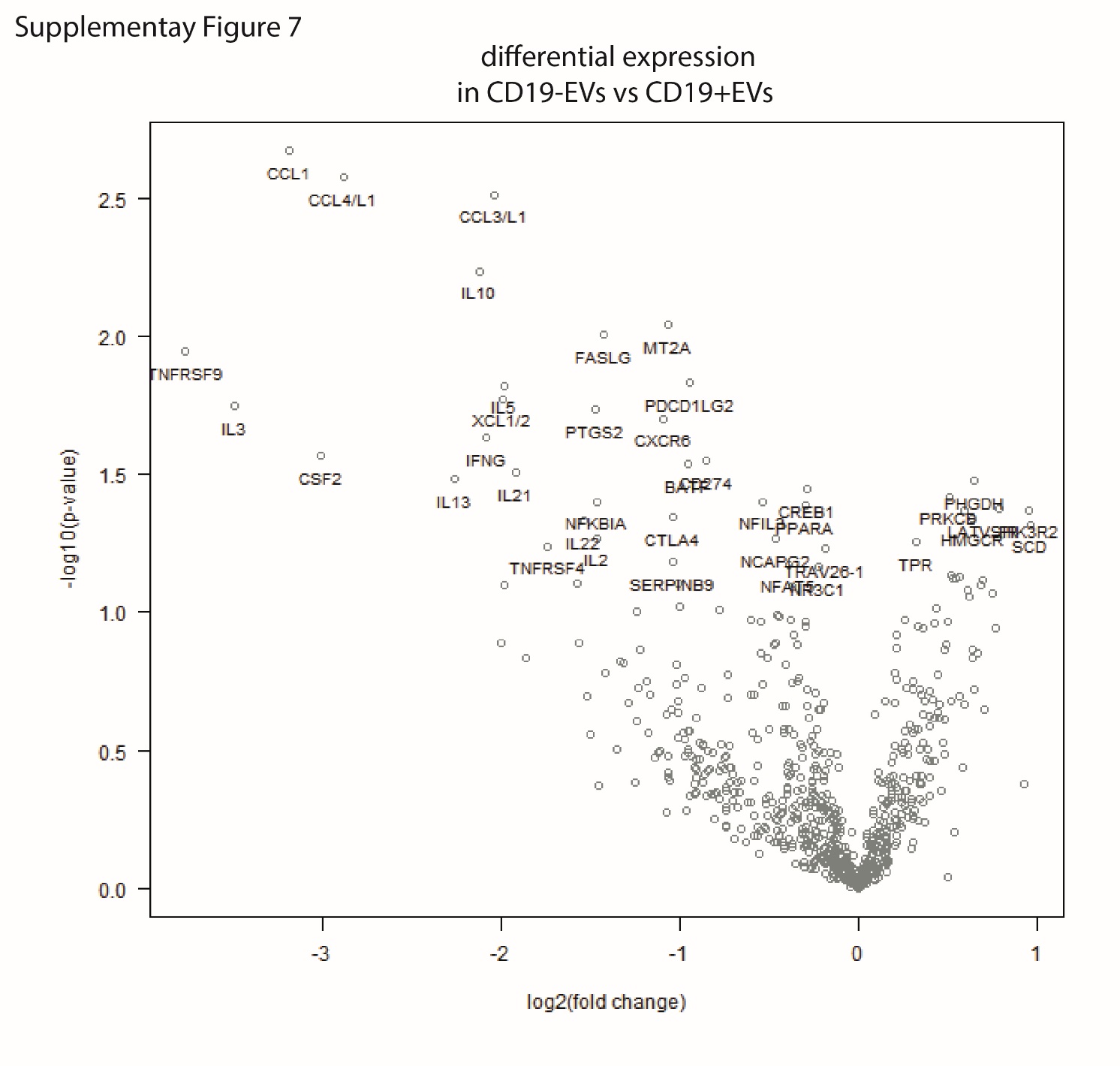


**Supplementary figure 7.** **Transcriptome analysis of CD19 CAR treated with CD19+EVs and CD19-EVs (n=4).** RNA-seq data plotted as a scatterplot of fold change of gene expression (x-axis) plotted against p.value (y-axis) where each dot represents single gene.

| **Antibody** | **Source** | **Identifier** |
| --- | --- | --- |
| anti-human CD4-FITC | Biolegend | Cat.# 317416 |
| anti-human CD8a-PE | Biolegend | Cat.# 300908 |
| anti-human GAPDH HRP | Abcam | Cat.# ab185059 |
| anti-human CD45RA-PE | Biolegend | Cat.# 304108 |
| anti-human CD62L-APC | Biolegend | Cat.# 304810 |
| anti-human CD3-APC | Biolegend | Cat.# 300312 |
| anti-human IgG Fc Cross-Adsorbed Secondary Antibody, DyLight™ 650 | Invitrogen | Cat.# SA5-10137 |
| CD19 CAR Detection Reagent, human | Miltenyi | Cat.# 130-129-550 |
| FITC-Labeled Human CD19 | Acro Biosystems | Cat.# CD9-HF2H2 |
| streptavidin-FITC | Biolegend | Cat.# 405201 |
| goat Anti-Rabbit IgG Antibody, HRP-conjugate | Sigma | Cat.# A0545-1ML |
| anti-human CD19-PE | Biolegend | Cat.# 982402 |
| anti-human CD63-APC | Sony | Cat.# 2365020 |
| anti-human CD81-FITC | Sony | Cat.# 2347520 |
| rabbit anti-human CD63 Polyclonal Antibody | Invitrogen | Cat.# PA5-78995 |
| mouse anti-human CD19-HRP | Abcam | Cat.# ab195896 |
| Hoechst 33342 | Sigma | Cat.# 14533 |
| anti-human PDL-1-APC | Miltenyi | Cat.# 130-122-816 |
| anti-human IL13R2a-FITC | R&D systems | Cat.# FAB614F |
| anti-human PD-1-APC | Miltenyi | Cat.# 130-122-169 |
| anti-human TIGIT-PE | Miltenyi | Cat.# 130-116-934 |
| anti-human CD57-APC-Cy7 | Miltenyi | Cat.# 130-111-811 |

**Supplementary table 1.** Antibodies used in flow cytometry analysis and Western blot.
